# Book review of Electrophysiological Recording Techniques, edited by Robert P Vertes and Robert W Stackman, Jr

**DOI:** 10.1186/1475-925X-10-63

**Published:** 2011-07-21

**Authors:** Vasil Kolev

**Affiliations:** 1 Institute of Neurobiology, Bulgarian Academy of Sciences, Acad. G. Bonchev str., bl. 23, 1113 Sofia, Bulgaria

## Abstract

The review presents the newly published book "Electrophysiological Recording Techniques" by Humana Press. This is an important collection of articles devoted to an issue that has gained increasing attention in modern neuroscience research - electrophysiological recording techniques. The present book is a timely update of methods spanning from single cell recordings to local field potentials, cortical, and scalp EEG recordings. The book presents the progressive development of electrophysiological recording methods by including both existing and new advanced technologies. Theoretical considerations on important issues like current source density analysis, local field potential and signal generation are also in focus. In most of the chapters, methods are illustrated with specific experimental results and are discussed towards future developments and applications. Chapters can be read independently because each chapter appears with its own theoretical background, literature survey and specific methodology. The book can be of interest to a broader audience willing to look at the contemporary state of development of electrophysiological methods. Also, it can be of special interest to scientists with high expertise working in the field of neuroscience and behavior.

## Introduction

The book "Electrophysiological Recording Techniques" published recently by Humana Press is an important collection of articles devoted to an issue that has gained increasing attention in modern neuroscience research. Several sources of this interest exist.

Since the discovery of biological signals in the brain, the question of the neural codes of information processing has remained a challenging one. Current models of information processing and communication in the brain recognize three different levels: (a) microscopic, where ion channels, spiking neurons and neuronal assemblies are the neural substrate of information processing and transmission; (b) mesoscopic, where oscillatory electroencephalographic (EEG) activity is detected at different scales reflecting the formation and functioning of distributed neural networks, and (c) macroscopic, where regional large-scale activation and synchronization support the integrative information processes. Electrophysiological techniques have made it possible to measure and analyze in detail the bioelectric signals at each level - in single neurons, in neuronal ensembles, and integrated spatially distributed neural structures. However, modern neuroscience still confronts the problem of how neural codes are transferred and communicate across different levels and how multi-scale information processing is organized. The collection of research issues in the current book is a contributing step to this long-standing question.

Neuroscience researchers were and still are attracted by the electrophysiologically recorded signals due to their major property - good time resolution, which allows one to describe precisely the temporal dynamics of neurophysiological events. Electrophysiological signals may be recorded with a time resolution of less than 1 ms, which makes it possible to follow both fast and slow processes of information processing. These signals can therefore capture with high fidelity a variety of neural mechanisms operating at different temporal scales.

The deviations in the organization and communication of the neural substrate revealed by bioelectric signals also have been addressed by both fundamental and clinical research as providing relevant basis to model pathological conditions. Thus, by developing electrophysiological recording methods, scientists approach a fundamental aim of neuroscience - to uncover, possibly in real-time, the mechanisms supporting the regulation, maintenance and perfection of vital, mental and cognitive functions in the organisms. A converging general aim is to understand how the brain works.

## Overview

The present book is a timely update of methods spanning from single cell recordings to local field potentials (LFPs), cortical, and scalp EEG recordings. The book presents the progressive development of electrophysiological recording methods by including both existing and new advanced technologies. Importantly, methodological progress is exposed not only theoretically, but also conceptually and in applied context by experimental validation. Along with technical details, new neurophysiological frameworks are suggested for neural organization and across-scale interaction during information processing in normal and pathological conditions. In this regard, newly emerging frames are outlined in animal models and the human brain. The book can be of interest to a broader audience willing to look at the contemporary state of development of electrophysiological methods. Also, it can be of special interest to scientists with high expertise working in the field of neuroscience and behavior.

The editors, Robert P. Vertes and Robert W. Stackman, Jr. selected a total of 11 articles which cover specific questions of modern electrophysiological recording techniques, analysis and applications. There are also theoretical considerations on important issues like current source density analysis, LFPs and signal generation. Most of the presented methods are illustrated by experimental results and supported by discussions towards future developments and applications. Chapters can be read independently because each chapter has its own theoretical background, literature survey and methodological description.

## Neuronal Recording Techniques

Most of the chapters in the book are devoted to neuronal recording techniques (single cell and multi-unit recordings, and multi-channel recordings of ensembles of neurons) that present the state of the art in the field:

Pinault describes a technique that permits the *discrete labeling of individual neurons *during simultaneous extracellular recording, which serves as a tool for defining the discrete connectivity of neurons whose physiological properties have been identified.

*New strategies applied to in vivo single-unit recordings *from freely moving rodents are presented in the chapters by Fenton et al., Kuang and Tsien, Hampson et al., and Stackman. The advantages of the new digital multichannel telemetry (DT) system over the analog wireless systems are shown by Fenton et al., together with an outline of two applications for DT - tetherless recordings from freely moving rodents during truly unrestricted behavioral performance and an epilepsy monitoring system for use in humans. The authors outline digital signal processing together with the basic principles of telemetry, describing how DT preserves signal fidelity while allowing subjects to move around in an unconstrained way.

Kuang and Tsien address two of the exciting challenges emerging in the field of neuronal investigations, that of how to acquire *high-density ensemble neuronal activity *from wild type and genetically engineered mice, and second, how to analyze these data. They describe a device which is capable of acquiring up to 128 channels of neuronal activity data simultaneously from freely moving mice. The decoding and deciphering of this real-time ensemble-recording technology offer a great promise for application to multiple brain regions making an impact of potential significance on the development of brain-machine interfaces.

In the chapter by Stackman, the *relation of neuronal firing patterns of limbic neurons with distinct behavioral sequences *are in research focus. Further, this chapter uses the information obtained from the rodent head direction cells to build a model to delineate the degree to which directional correlates of single-unit activity relate to spatial navigation. The authors provide also a comprehensive overview of current approaches for defining the relationships between the firing patterns of groups of neurons recorded from freely behaving rodents. Finally, suggestions for alternative experimental approaches that might better address brain-behavior relationships are proposed.

## Local Field Potentials

The chapters of Leung and Chen et al. are devoted to theoretical problems of LFP generation and analysis by means of current source density (CSD) method, together with applications to real signals. Leung describes the basic principles of field potential recordings and CSD analysis. Using experimental data as well as model systems of hippocampal pyramidal cells, Leung convincingly illustrates the differential patterns of current flow along pyramidal cell dendrites and soma to the activation of different segments of the neuron (basal dendrites, soma, proximal or distal regions of apical dendrites), depicting averaged evoked potentials and their derived CSD profiles.

Chen et al.'s investigations complement Leung's chapter and describe an *in vivo *procedure for CSD analysis of ongoing, non-triggered neural activity which is a contribution to this field of research. A method termed "phase realigned averaging technique" (PRAT) is described which extracts low amplitude signals from continuous streams of activity recorded from the cortical fields in awake animals. The PRAT method allows for a determination of the relationship of endogenous activity (e.g., alpha rhythm) to behaviorally relevant events, such as sensory or motor responses to external stimuli.

## EEG and Event-related Potentials

Bressler describes theoretically the principal methods for event-related potential recordings and analysis. These techniques are applicable for studying LFPs recorded with depth (intracortical) electrodes as well as signals from the scalp (e.g., EEG recordings in humans). Main principles of analysis in time, frequency and spatial domains are described in brief. The so-called Granger causality method illustrates the interdependency analysis between two (or more) signals.

## Multi-scale Approaches

The book presents a plenty of results form behavioral experiments, supplying the literature with newly acquired data related to neuronal encoding and behavioral correlates of neuronal oscillations.

The chapter by Albo et al. describes current methods for unit-field (and field-field) analysis, with the application of spike-field coherence techniques for studying of unit-field oscillations. The chapter provides a comprehensive overview of various approaches to assessing functional interactions among synchronously occurring signals (spike trains and field potentials) across the brain. As a direct application of some of the described techniques, they present findings showing a three way interaction (coherence) between theta rhythmic units in the anterior thalamus and theta oscillations in the hippocampus and retrosplenial cortex, suggesting that hippocampus may drive the anterior thalamus, which in turn rhythmically paces the retrosplenial cortex, with implications for the role of theta rhythms in limbic functions.

By means of recordings from ensembles of hippocampal neurons, Hampson et al. describe a "closed loop system" which distinguishes among the separate behavioral components in a two choice delayed nonmatch to sample (DNMS) task, and then uses ensemble activity at phases of the task to both predict choice behavior and modify it during task performance. In effect, ensemble activity (or codes) was used to adjust delay times (between sample and choice) during ongoing trials to improve performance *on those trials*. Specifically, depending on the relative strength (or efficacy) of the ensemble code in the sample phase of the task, the delay between sample and nonmatch task phases could be shortened or lengthened, thereby improving performance. It is well recognized that the septum and hippocampus are strongly interconnected and together serve as a functional unit generating the hippocampal theta rhythm. Theta serves a well-recognized role in mnemonic functions.

In a major advance in examining septo-hippocampal interactions, Goutagny et al. developed a remarkable *in vitro *preparation in which the septum and hippocampus are simultaneously dissected (with connections between them intact) and kept viable for at least 8 h. In addition, with a barrier placed between them, the two structures can be independently manipulated to assess the effects of one on the other. Using this preparation, Goutagny et al. confirmed the pronounced septal influence on the hippocampus in the modulation of theta, and further showed that hippocampal theta activity, in turn, exerts a strong driving influence on the septum.

## Clinical Applications

Mayberg and Holtzheimer provide a comprehensive survey on imaging studies that led to the use of deep brain stimulation (DBS) for treatment of the major depressive disorder (MDD). It was found that certain regions of the frontal cortex, such as the subcallosal cingulate cortex (SCC) were hyperactive in MDD, whereas other areas such as dorsolateral prefrontal or posterior cingulate cortices were hypoactive in MDD, and that successful antidepressant treatment normalized activity in these regions. This suggested a critical role for the frontal/prefrontal cortex (particularly the SCC) in MDD. Mayberg and Holtzheimer describe in detail the specific procedures used for DBS of the SCC and summarize the extremely promising results that have been obtained to date with the technique with two groups of patients with treatment resistant depression. DBS is not only a cutting edge procedure for the treatment of depression, but also used in conjunction with other methods and has the potential to define an extended circuitry responsible for MDD.

## Conclusion

The book "Electrophysiological Recording Techniques" edited by Vertes and Stackman was published recently to enrich our knowledge on specific electrophysiological recording methods for investigation of neuronal and field activity in the brain. This is an important state-of-the-art collection of articles presenting the most advanced developments in the field. I would recommend the book to everybody who is interested in the contemporary techniques for recording and analysis of electrophysiological signals and their application to studying brain and behavior.

## Competing interests

The author declares that he has no competing interests.

